# Biliary microbiota in disease-free, obstructive and post-drainage biliary tracts

**DOI:** 10.3389/fcimb.2025.1674341

**Published:** 2025-12-10

**Authors:** Guiyuan Zhang, Linyuan Zeng, Bin Chen, Haitao Dai, Keyu Tang, Ruotong Huang, Xianhong Xiang, Jianyong Yang, Juhua Yang, Xiuling Song, Yi Ma, Run Lin, Yonghui Huang

**Affiliations:** 1 Department of Radiology, The First Affiliated Hospital, Sun Yat-sen University, Guangzhou, Guangdong, China; 2 Center for Precision Medicine, Sun Yat-Sen University, Guangzhou, Guangdong, China; 3 Department of Medicine, University College London, London, United Kingdom; 4 Vision Medicals Co., Ltd., Guangzhou, China; 5 Department of Organ Transplantation, The First Affiliated Hospital, Sun Yat-sen University, Guangzhou, China

**Keywords:** biliary microbiota, bacterial communities, PTCD=percutaneous transhepatic cholangial drainage, NGS - next generation sequencing, biliary drainage, biliary infections

## Abstract

**Introduction:**

Despite years of research, knowledge about the microbial populations of human physiological bile has remained limited. Bile sampling techniques, such as Endoscopic Retrograde Cholangiopancreatography (ERCP), percutaneous biliary drainage, and intra-operative sampling, are invasive procedures typically performed only in the presence or suspicion of biliary tract disease. Furthermore, the increased incidence of bacterial infections following biliary drainage poses a significant clinical concern; however, the relationship between biliary drainage and biliary flora remains poorly understood. In this study, we present a distinct taxonomic composition of bacterial communities identified in bile samples from disease-free individuals, as well as from obstructive and post-drainage biliary tracts.

**Methods:**

A metagenomic sequence analysis of bile samples from patients with MBO who underwent percutaneous biliary drainage (PTBD) at our center from 1st May 2021 to 1st March 2022, which were divided into 2 groups, as the MBO group (n = 29) and BD group (n = 27). Eight liver donors were included as a control group.

**Results:**

Abundant bacterial populations were detected in the bile of liver donors, revealing a highly similar microbial composition in both disease-free and malignant obstructive biliary trees. Notably, biliary drainage was found to alter the composition of bile microbiota, resulting in decreased microbial diversity and an association with an increase in antibiotic resistance genes.

**Discussion:**

These findings provide fundamental knowledge on the composition of the human bile microbiota and present new evidence to support that biliary drainage induces a shift in bile microbiota, rendering it more aggressive and resistant to antibiotics.

## Introduction

Although bile has traditionally been regarded as bacteriologically sterile in healthy individuals (Nielsen and Justesen, 1976), this perspective has been consistently challenged. Flemma et al. (1967) observed that a consistent number of patients had a positive bile culture without having had any signs, symptoms or history of cholangitis. They hypothesized that bacteria could exist in bile without causing any symptoms attributable to their presence and named this condition “asymptomatic bactibilia”in 1967 (Flemma et al., 1967). About 40 years later, the introduction of 16S ribosomal RNA sequencing confirmed the presence of microbes in bile samples that were previously considered sterile by culture-based techniques (Swidsinski et al., 1995). In the same time, the concept of the human biliary microbiota was introduced (Swidsinski et al., 1995). In contrast to the well-established understanding of gut microbiota developed over the last decade, the characterization of the human bile microbiota has been hampered by difficulties in accessing biological samples and the lack of adequate methodologies to assess molecular studies (Nicoletti et al., 2020). The invasive nature of procedures to collect bile samples, such as endoscopic retrograde cholangiopancreatography (ERCP) or percutaneous interventions, poses risks to individuals (Nicoletti et al., 2020). Additionally, ethical considerations regarding research on normal human bile limit the scope of specific research activities. Consequently, very little is known about the human physiological biliary microbiota.

Since 2017, when our center reported the world’s first case of non-ischemic liver transplantation (He et al., 2018), this technique has been routinely performed at our facility. During the procedure, the donor liver is continuously perfused with a normothermic machine, ensuring its viability for procurement, preservation, and transplantation while still producing bile (He et al., 2018). This approach has enabled us to obtain bile samples from disease-free biliary tracts. The knowledge about the composition of the biliary microbiota in health represents the first step in the understanding of the influence of the microbiota on the development of biliary diseases.

Percutaneous transhepatic biliary drainage (PTBD) is a minimally invasive technique widely to manage biliary obstruction and obstructive jaundice, boasting a high technical success rate and definite therapeutic efficacy (Kapoor et al., 2018). However, over 40% of patients experience drainage-related infectious complications, such as sepsis, cholangitis, abscess, or cholecystitis, within one month after PTBD. Once these complications occur, the 30-day inpatient mortality rate approaches 30% (Sameer and Mishra, 2021). Elevated infection rates following biliary obstruction and drainage represent a widely observed phenomenon. Some researchers attribute this to bacterial colonization in the obstructed biliary tract (Lee, 2009); however, the precise causal relationship between these factors remains unverified by empirical evidence.

Previous studies have frequently concentrated on the rapid identification of pathogenic bacteria, yet have not adequately addressed the comprehensive changes within the entire microbial community. Little is known about whether biliary microbiota dysbiosis in obstructive biliary tracts is connected with infections or correlated to the complex bacterial colonization associated with drainage. Therefore, gaining a deeper understanding of the bacterial communities in disease-free biliary systems compared to those in obstructed biliary systems, as well as exploring the relationship between drainage interventions and biliary microbiota, is crucial for developing effective prevention and treatment strategies for biliary infections. Traditional culture techniques for identifying bacteria bears many shortcomings, including proliferate bias due to laboratory conditions, underestimation of the bacterial diversity, and time-consuming (Nicoletti et al., 2020; Gu et al., 2021). Thus, the development of facile and robust strategies for investigating the biliary microbiome comprehensively is eagerly awaited. Recent advances in molecular techniques (Tajeddin et al., 2016), particularly next-generation sequencing (NGS) (Gu et al., 2019), are beginning to shed light on the composition and functional characteristics of the biliary microbiota. These innovative methodologies allow for a more comprehensive analysis of microbial diversity and the relationship of biliary microbiota to various biliary diseases, including cholangitis, biliary obstruction, and even conditions like primary sclerosing cholangitis (Pereira et al., 2017; Gu et al., 2019; Petrov et al., 2020; Song et al., 2020; Wensel et al., 2022).

In this study, we utilized NGS technology to analyze bile samples from eight liver transplant donors without biliary pathology, as well as bile samples from 56 patients who underwent PTBD treatment due to malignant tumor-induced biliary obstruction. Our aim was to gain a comprehensive understanding of the microbial composition and characteristics in disease-free biliary tracts, obstructed biliary tracts, and post-drainage biliary tracts. Notably, we collected bile not only from normal gallbladders but also obtained fresh bile secreted by the liver through the common bile duct, aided by non-ischemic liver transplantation techniques.

## Methods

### Patient enrollment and samples collection

The protocol of this study was reviewed and approved by the Ethics Committee of the First Affiliated Hospital, Sun Yat-sen University (No. 2022-528). Informed consent forms were obtained from all patients. Patients with malignant biliary obstruction who were referred to our department and scheduled to receive percutaneous biliary drainage (PTBD) from May 2021 to March 2022, were enrolled. Patients were excluded from this study if they (1) were ineligible or refused to sign informed consent, (2) had any contraindications or were unable to complete the PTBD procedure, (3) had a long-term use of antibiotics or other treatments that may affect the results of this study, (4) had an expected survival of less than three months, or (5) had any other issues that unsuitable for this study as determined by researchers.

All the PTBD and bile sample collection procedures were performed under sterile condition. The distal end of drainage catheter was placed within the dilated intrahepatic bile duct using the percutaneous transhepatic puncture technique. After catheter placement, bile samples were immediately collected aseptically from the proximal end of biliary drainage tube. Samples collected from 29 patients who underwent initial PTBD procedures due to malignant biliary obstruction were assigned to MBO group. Samples obtained from 27 patients who received a re-intervention over two week after initial biliary procedure including PTBD or internalized stent were assigned to biliary drainage (BD) group. The bile sample of each patient was divided into two parts for conventional microbiological detection and mNGS, respectively.

Liver transplantations were performed in Class I operating room, which requires that the number of dust particles per cubic meter of air is less than 100. Bile samples from eight donor livers without biliary pathology were collected during these procedure. Among them, four bile samples were collected as fresh secretions from the common bile duct with the assistance of a non-ischemic liver transplantation technique, while another four samples were obtained directly from gallbladder aspiration during the standard liver transplantation process. These samples were only detected by mNGS and served as the control group.

### DNA extraction and sequencing

Bile samples were sealed and placed in a foam box containing an ice pack and immediately transported to commercial laboratory (Vision Medicals, China) for mNGS texting. Once the laboratory received the bile samples, samples processing, DAN extraction, high-throughput sequencing according to the laboratory’s standard operating procedures (Li et al., 2022). Total DNA samples were extracted using the QIAamp^®^ UCP Pathogen DNA Kit (Qiagen, Hilden, Germany) according to the manufacturer’s instructions. Human DNA was removed using Benzonase (Qiagen, Hilden, Germany) and Tween20 (Sigma, U.S.) (Amar et al., 2021). Libraries were constructed using the Nextera XT DNA Library Prep Kit (Illumina, San Diego, CA, U.S.) (Miller et al., 2019). Library quality was assessed using the Qubit dsDNA HS Assay kit and the High Sensitivity DNA kit (Agilent, U.S.) on the Agilent 2100 Bioanalyzer. Library pools were then sequenced using the Illumina Nextseq 550Dx sequencer for 75 cycles of single-end sequencing, generating approximately 20 million reads per library. Negative controls were prepared using peripheral blood mononuclear cell (PBMC) samples from healthy donors, with 10^5^ cells/mL prepared in parallel with each batch using the same protocol. Sterile deionized water was extracted alongside the specimens to serve as non-template controls (NTC) (Li et al., 2018; Miller et al., 2019).DNA-free water was used as a blank control group to assess the degree of background contamination associated with the DNA extraction kit and sequencing reagents.

### Bioinformatics analyses

High quality data were obtained by Trimmomatic to remove low quality reads, adapter contamination, duplicate reads, and reads shorter than 50bp (Xu et al., 2020). Low complexity reads were removed by Complexity with default parameters (Bolger et al., 2014). Human sequence data were identified and excluded by mapping to a human reference genome (hg38) using Burrows-Wheeler Aligner software (Song et al., 2020). We designed a set of criteria similar to the National Center for Biotechnology Information (NCBI) criteria for selecting representative assembly for microorganisms (bacteria, viruses, fungi, protozoa, and other multicellular eukaryotic pathogens) from the NCBI Nucleotide and Genome databases. Pathogen lists were selected according to Johns Hopkins ABX Guide (https://www.hopkinsguides.com/hopkins/index/Johns_Hopkins_ABX_Guide/Pathogens), Manual of Clinical Microbiology, and clinical case reports or research articles published in current peer-reviewed journals. The final database consisted of about 13000 genomes. Microbial reads were aligned to database with Scalable Nucleotide Alignment Program (SNAP v1.0 beta.18). A positive detection was reported for a given species or genus if the reads per million (RPM) ratio, or RPM-r was ≥5, where the RPM-r was defined as the RPMsample/RPMNC (i.e., the RPM corresponding to a given species or genus in the clinical sample divided by the RPM in the negative control) (Miller et al., 2019). In addition, to minimize cross-species misalignments among closely related microorganisms, we discounted the RPM of a species or genus that appeared in non-template controls and shared a genus or family designation that a penalty of 5% was used for species (Gu et al., 2021). Sequencing reads, genomic coverage, the relative abundance of each organism, and drug resistance genes were used for the next analyze. The site-by-species counts and relative abundance tables were input into R-base V.4.1.0 for statistical analysis. The structures of the biliary microbial communities from the three groups were examined, and different categories of the sampled biliary tract were characterized by distinct compositions of metagenomic reads on high taxonomic (genus and species) levels. The observed discrimination, from a taxonomic point of view, was confirmed by calculating the relative abundances of bacterial species presented in each sample using an approach based on the identification and quantification of marker genes. Alpha diversity of the microbiota profile for each subject was assessed by groups at different (genus and species) levels of data using the Vegan package in R (version 2.5.7). Beta diversities were calculated over taxonomic profiles using the Bray-Curtis distance as implemented in the Vegan R package. We calculated the Bray-Curtis distance at genus-level abundance to reflect the similarity among bile samples of different groups. If the beta diversity index of Bray-Curtis distance is shorter, means the community of bile microbiota between samples was similar. Principal component analysis (PCA) ordinations and Principal Coordinates Analysis (PCoA) were used to visualize the clustering of samples based on their genera or species-level compositional profiles. PCoA ordinations was stated by the function of dudi.pco in ade4 package in R (version 1.7.18). Different groups were assessed using permutational multivariate analysis of variance (PERMANOVA) and PCA stat with the Vegan package in R (version 2.5.7), *p* value<0.01 was considered statistically significant. We tried to identify some signature microbes at the higher taxonomic hierarchy levels (genus and species) in 3 groups. LEfSe (linear discriminant analysis of the effect size) analysis was applied to identify genera and species with significant differences in abundance between groups. We identified differential distributions of genera and species using the default cut-offs (LDA score >3.0; P value <0.05).

Antibiotic resistance genes (ARGs) were identified by comparing contigs against the Comprehensive Antibiotic Resistance Database (CARD). In order to analyze the antibiotic resistance gene content, four different pipelines were used for ARG quantification. Subsampled quality-controlled reads were processed with deepARG using CARD v.1.05 as a reference database to define the composition and abundance of ARGs for each sample (Arango-Argoty et al., 2018). This database included known molecular sequences curated by human experts and mutations conferring resistance to antibiotics with clinical relevance. ARGs are classified into ARG families (genes with similar function) and drug classes (types of antibiotics targeted by ARGs).

### Bile culture and antibiotic resistance testing

Bile samples from the MBO group and BD group underwent bacterial culture and antibiotic resistance testing. Bile cultures were tested by the VITEK2 Automatic Rapid Microbial Identification Intelligent Analysis system, and its supporting identification card. Recommendations and criteria from Clinical and Laboratory Standards Institute (CLSI) and European Committee on Antimicrobial Susceptibility Testing (EUCAST) were used to define susceptibility or resistance to antibiotic agents.

### Statistical analysis

SPSS Statistics 22 was utilized to conduct statistical analysis on the comparison of clinical variables between groups. The results for categorical variables were presented as absolute and relative frequencies, while the results for continuous variables were expressed as means and standard deviations (SD) if they followed a normal distribution, or as medians and interquartile ranges (IQR) if the distribution was not normal. Chi-squared test was performed for all categorical variables. For continuous variables, two independent-sample t-test was conducted for normally distributed continuous variables and Mann–Whitney U test was conducted for those that were not normally distributed. Correlation analysis were performed using Spearman’s correlation analysis and *p* value<0.05 was considered statistically significant.

## Results

### Biliary drainage increased the infection rate of malignant biliary obstruction

A total of 56 patients with biliary tract malignancies and 8 liver donors were included in this study. Specifically, 29 bile samples were collected for the MBO group, while 27 bile samples were designated for the BD group. No significant differences regarding gender distribution and obstruction sites of the biliary tract were found between the groups. On the other hand, patients in the BD group were younger, with lower serum bilirubin levels, and better liver function, but experienced more frequent febrile and antibiotic use, as well as a higher rate of positive bile cultures. And bile duct dilatation was more pronounced in the MBO group. The clinical characteristics of the patients are summarized in **Supplementary Table S1** (**Supplementary File**).

Next-generation sequencing (NGS) and bile cultures were performed on each sample. According to the culture results, bacterial infections were detected in 70.4% (19/27) of the BD group samples and 13.8% (4/29) of the MBO group samples (**Table 1**).

**Table 1:** Results of positive bile cultures and resistance to antibiotic agent of strains.

Sample	Strain	β-lactam antibiotics															Aminoglycoside	Tetracyclines		Macrolide	Lincosamides	Quinolones		Sulfonamide
		PG	OX	AMP	KZ	CXM	CTX	CRO	CAZ	FEP	FOX	AMC	SAM	SCF	TZP	ATM	CN 10	TE	T CG	E	CLIN	CIP	LEV	SXT
BD 1	*E.coli*			R									R										R	R
BD 4	*KP*			R																				
BD 6	*E.cloacae*			R	R	R	R		R		R	R	R	R	R	R			R					R
	*AB*																							
	*CA*																							
BD 8	*S. epidermidis*	R																						
BD 9	*E.coli:ESBL(+)*			R	R	R	R	R		R			R			R							R	R
	*PM*																							
BD 10	*A.* *hydrophila*																							
BD 11	*S. epidermidis*	R	R																	R				
BD 13	*S. aureus*	R																		R	R			
	*AB*																							
	*SA*																	R						
BD 14	*S.* *epidermidis*	R																						
BD 16	*AB*	R																						
	*S.aureus*																							
BD 19	*E. faecium*	R		R																R			R	
	*PA*																					R	R	
	*CA*																							
BD 20	*KP*																							
	*E.faecium*																							
BD 21	*E.faecium*	R		R																			R	
	*EA*																			R				
BD 22	*Cpd*																							
BD 23	*KP*			R	R								R				R							R
	*PM*																		R					
BD 24	*S.aureus*	R																		R				
BD 25	*VV*			R																				
	*KP*																							
BD 26	*A.lwoffii*																							
BD 27	*ECL*				R						R	R												
BO 5	*S.salivarius*																							
BO 23	*SA*																	R		R	R		R	
BO 27	*E.coli*			R	R								R											
BO 29	*E.faecalis*																							
MIC(ug/ml)		>=.05	>=4			>=64	>=64	>=64	>=64	>=16	>=64	>=8	>=32	>=64	>=128				>=8	>=8	>=0.5	>=2	>=320	
KB(mm)				6	6											9	6	16						8

- **R**: Resistant.

- Empty cells: Susceptible or not tested.

-PG, Penicillin G; OX, oxacillin; AMP, ampicillin; KZ, cefazolin; CXM, cefuroxime; CTX, cefuroxime axetil; CRO, ceftriaxone; CAZ, ceftazidime; FEP, cefepime; FOX, cefoxitin; AMC, amoxicillin/clavulanate; SAM, ampicillin/sulbactam; SCF, cefoperazone/sulbactam;

TZP, piperacillin/tazobactam; ATM, aztreonam; CN10, gentamicin; TE, tetracycline; TCG, tigecycline; E, erythromycin; CLIN, clindamycin; CIP, ciprofloxacin; LEV, levofloxacin; SXT, compound sulfamethoxazole (sulfamethoxazole/trimethoprim).

-*E.coli, Escherichia coli; KP, Klebsiella pneumonia; E.cloacae, Enterobacter cloacae; AB, Acinetobacter baumannii;CA, Candida albicans; S. epidermidis, Staphylococcus epidermidis; PM, Pseudomonas hydrophilic; A.hydrophila, Aeromonashydrophila; S.aureus*,

*Staphylococcus aureus; SA, Streptococcus anginosus; E. faecium, Enterococcus faecium; PA, Pseudomonas aeruginosa;CA, Candida albicans; EA, Enterococcus avium; Cpd, Corynebacterium pseudodiphtheriticum; VV, Vibrio mimicus; A.lwoffii, Acinetobacter lwoffii; ECL*,

*Enterobacter cloacae; S.salivarius, Streptococcus salivarius; SA, Streptococcus sanguis.*

-MIC, Minimum inhibitory concentrations; KB, diameter of inhibition zone.

A total of 35 bacterial isolates were obtained from 56 bile samples; however, polymicrobial infections were only identified in 33.3% (9/27) of the BD group samples. The most frequently isolated bacterium was *Klebsiella pneumoniae* (4/35), followed by *Acinetobacter baumannii* (3/35), *Enterococcus faecium* (3/35), *Escherichia coli* (3/35), *Staphylococcus aureus* (3/35), and *Staphylococcus epidermidis* (3/35). In BD group, the polymicrobial infections involved various combinations, including *Escherichia coli* and *Proteus mirabilis* in one sample; *Acinetobacter baumannii* and *Staphylococcus aureus* in another; Klebsiella pneumoniae and *Enterococcus faecium* in one; *Enterococcus faecium* and *Enterococcus avium* in one; *Klebsiella pneumoniae* and *Proteus mirabilis* in one; *Klebsiella pneumoniae* and *Vibrio mimicus* in one; *Enterobacter cloacae*, *Acinetobacter baumannii*, and *Candida albicans* in one; *Staphylococcus aureus*, *Acinetobacter baumannii*, and *Streptococcus anginosus* in one; and *Enterococcus faecium*, *Pseudomonas aeruginosa*, and *Candida albicans* in one.

Antibiotic susceptibility testing was conducted for all cultured bacteria (**Table 1**). All *Escherichia coli* strains exhibited resistance to ampicillin and ampicillin/sulbactam. All *Enterobacter cloacae* strains demonstrated resistance to cefazolin and amoxicillin/clavulanate. Moreover, all *Staphylococcus epidermidis* strains were resistant to Penicillin G. Notably, 20.0% of the strains exhibited multidrug resistance (MDR) phenotypes, including two *Escherichia coli* strains, one *Enterococcus* spp., one *Enterobacter* spp., one *K. pneumoniae*, one *Staphylococcus* spp., and *one Streptococcus* spp. Additionally, one *Escherichia coli* strain tested positive for extended-spectrum β-lactamases (ESBL).

### The disease-free biliary tract harbors a stable and diverse microbiome

The bacterial microbiomes of bile samples from the control, MBO, and BD groups were analyzed. In the control group, eight bile samples were aseptically collected from donor livers with no pathology during surgery. Four samples were obtained through direct aspiration from the gallbladder of donor livers, the other four samples were collected from the common bile ducts of the donor livers during ischemia-free liver transplantation, as the blood supplying of the donor lives were not stopped and still continuously secreted bile. The DNA extracted from the bile of these eight liver donors was analyzed using NGS.

**Figure 1:**
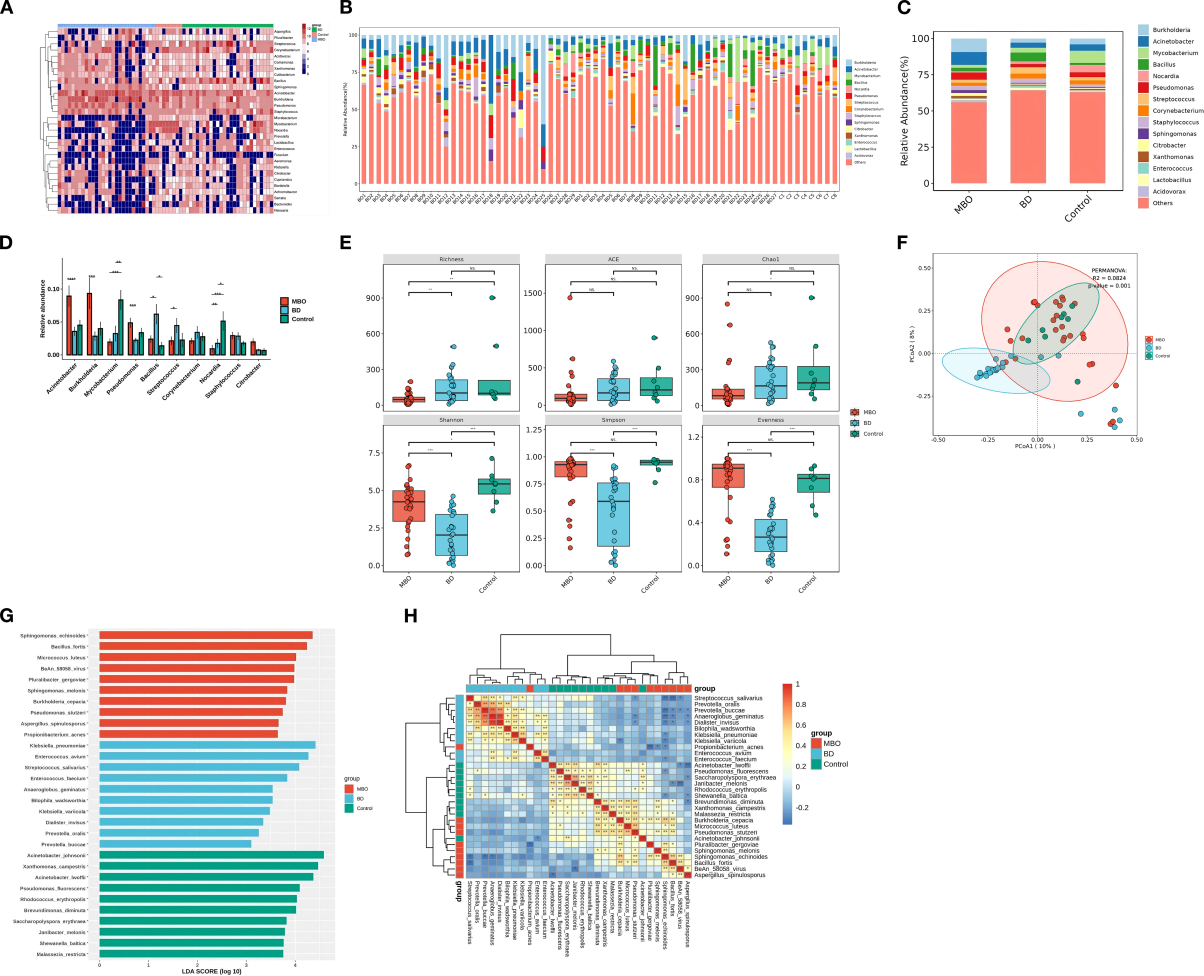
The composition of biliary microbiota in different groups. **(A)** Distribution and abundance of bile microbiota at the genus level in each sample The brown areas represent bacteria enrichment and the blue areas represent bacteria deletion. Genomes assigned to the genus of *Acinetobacter* were highly prevalent and ubiquitous in almost bile samples. **(B)** Genus composition of each samples. **(C)** Distribution and abundance of bile microbiota at genus level among three groups. **(D)** The relative abundance of top 10 genera of microbial communities in three groups. **(E)** Microbial diversity with individual samples (α-diversity) among three groups. Significant differences were obtained between BD group and MBO group (p value<0.001) as measured with common indices Shannon, Simpson and Evenness, which no statically significant differences were obtained between Control group and MBO group. MBO group had a significant reduce in richness compared to other groups (p value<0.01). **(F)** β-diversity differs significantly between BD and other groups in PCoA plot and NMDS plot (*p* value <0.001). Clustering of biliary samples based on Unifrac PCoA analysis of bacterial metagenomics. Each point represented a sample. Groups are color coded and distance between two points indicates differences in community composition, with shorter distances indicates smaller differences. The β-diversity of microbial communities in control group and MBO group were similar (*p* value =0.053 >0.001). **(G)** Barplots showing the LDA (linear differentiation analysis) scores for the bacterial species that are considered to be discriminative among the three groups of bile samples. **(H)** Heatmap showing the distribution of the biomarker bacterial species, the brown areas represent species enrichment and the blue areas represent species deletion. *p<0.05,**p<0.01,***p<0.001.

In addition to the detection of the commonly found *Acinetobacter* and *Burkholderia* genera in the biliary system, the control group exhibited a notable proportion of *Mycobacterium* and *Nocardia* genera in its bacterial community (**Figures 1A–D**). The alpha diversity, measured by the Shannon index, was significantly higher n the control group at 5.31 (standard deviation [SD]=1.00) compared to the other two groups (**Figure 1E**). Furthermore, the Bray-Curtis dissimilarity index for intra-group analysis was 0.28 (SD=0.12), the lowest among the three groups (**Figure 1F**). These suggest that the disease-free biliary tract harbor a stable and diverse microbiome.

### The richness of microbial community is significantly reduced in malignant obstructive biliary tracts

The relative abundances of *Burkholderia*, *Acinetobacter*, *Pseudomonas*, and *Staphylococcus* increased in the MBO group while the *Mycobacterium* and *Nocardia*, genera were depleted in the MBO group. Genomes assigned to the genera of *Burkholderia* and *Acinetobacter* were ubiquitous in all the bile samples (**Figures 1A–C**).

In the MBO group, the relative abundances of *Burkholderia*, *Acinetobacter*, *Pseudomonas*, and *Staphylococcus* increased, while the relative abundances of *Mycobacterium* and *Nocardia*, which had been present in substantial proportions in disease-free biliary tracts, decreased significantly (**Figure 1D**). Compared to the control group, the Richness index in the MBO group showed a significant decline (P < 0.01), whereas the differences in the ACE index, Simpson index, Shannon index, Chao1 index, and Evenness index were not statistically significant (P > 0.01) (**Figure 1E**).

The genomes associated with *Burkholderia* and *Acinetobacter* were ubiquitous in all bile samples, and their relative abundances rose rapidly due to a consistent decrease in the relative abundances of other genera (**Figure 1D**). At this point, the intra-group dissimilarity was 0.31 (SD=0.11), indicating that the community structure remained relatively stable (**Figure 1F**). Additionally, LEfSe analysis identified *Burkholderia*, *Sphingomonas*, *Methylobacterium*, *Bacillus*, *Escherichia*, *Microbacterium*, and others as characteristic genera of the MBO group, with an LDA score greater than 3.0 (**Figure 1G**).

### Biliary drainage correlates with reduced microbial diversity

Compared to the MBO group, the relative abundances of *Acinetobacter*, *Burkholderia*, and *Pseudomonas* in the BD group were significantly reduced, while the relative abundances of some genera, such as *Bacillus* and *Streptococcus* increased significantly, resulting in a notable rebound in the richness index (**Figures 1A–E**). And the relative abundances of *Mycobacterium* and *Nocardia*, which decreased in MBO group, also increased significantly. Conversely, the diversity indices, including the Shannon index, Simpson index, and Evenness index, significantly decreased compared to the other two groups, while the differences in the ACE index and Chao1 index were not statistically significant (**Figure 1E**). These findings indicate that the BD procedure leads to a significant reduction in the microbial/bacterial diversity of the biliary microbiome.

Beta diversity analysis revealed that the bacterial community structure in the BD group was distinctly different from control group and MBO group, a complete discrimination between the BD group and other groups could be observed according to Principal Coordinates Analysis (PCoA) ordinations with an intra-group dissimilarity value (Bray-Curtis distance) of 0.53 (SD=0.39) (**Figure 1F**), suggesting substantial variability in the community structure within the BD group. This indicates that the BD procedure disrupts the balance of the biliary microbiome.

Notably, the characteristic genera of the BD group included *Staphylococcus*, *Klebsiella*, *Enterobacter*, *Aeromonas*, *Pseudomonas*, *Anaerococcus*, *Diplococcus*, *Campylobacter*, and *Mycobacterium*. Furthermore, there was a competitive relationship between the characteristic genera of the BD group and those of the MBO group (**Figures 1G, H**).

### Drainage correlates with distribution of antibiotic resistance genes in biliary microbiome

**Figure 2:**
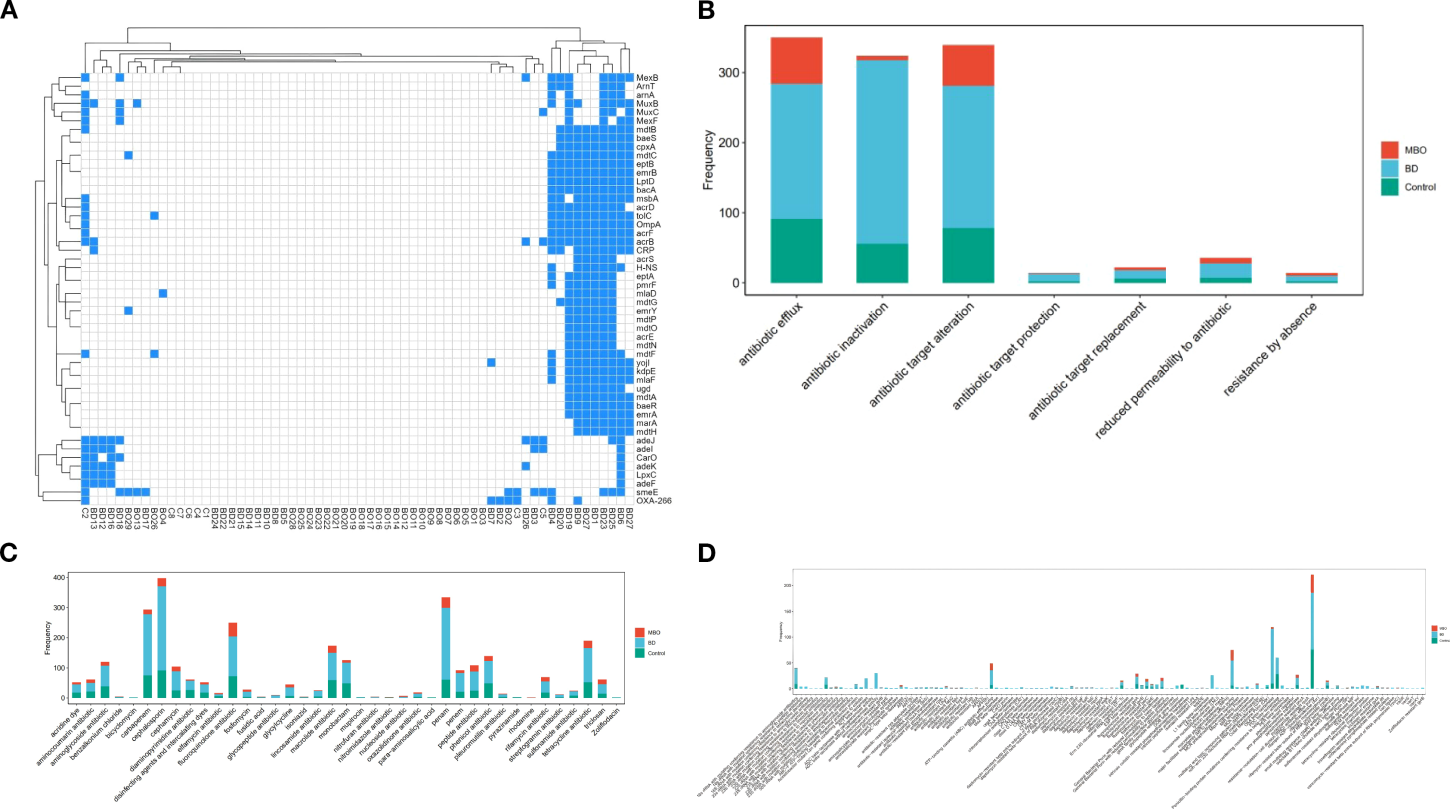
The antibiotic resistance genes of the biliary microbiota in different groups. **(A)** Distribution of ARGs among bile samples. Most of the resistance genes were present in BD group and could be associated with the prior procedure: BD1, BD4, BD6, BD9, BD19, BD20, BD23, BD25 had received biliary stent placement, BD27 had received external drainage combined with internal drainage. **(B)** Bar chart showing the number of detected ARGs for different antibiotic mechanisms in bile samples from control (green),MBO (red) and BD (blue) group. **(C)** Bar chart showing the number of detected ARGs for different antibiotic drug class in bile samples from control (green),MBO (red) and BD (blue) group. **(D)** Bar chart showing the number of detected ARGs for different AMR family in bile samples among control (green), MBO (red) and BD (blue) group. The total largest number of ARG was found in RND antibiotic efflux pump family.

The BD group exhibited more diverse and frequency of ARGs compared to other groups (**Figure 2**). A total of 727 ARGs subtypes were ascertained in the samples. The most frequent ARGs were *Enterococcus faecium* EF-Tu mutants conferring resistance to GE2270A, *Escherichia coli* rpoB mutants conferring resistance to rifampicin, *Staphylococcus* mutant fusA gene conferring resistance to fusidic acid, and *Staphylococcus aureus* rpoC conferring resistance to daptomycin. Additionally, the most abundant genes were mdtF, acrB, acrF, and mdtB, which belong to resistance-nodulation-division superfamily (RND) and encode outer membrane protein (OMP) relative to multidrug efflux pumps (**Figure 2D**). The most common mechanisms of antibiotic resistance in all three groups were consistent with antibiotic efflux, antibiotic inactivation, and alteration of antibiotic target (**Figure 2B**). We observed that the most abundant and numerous ARG types were found in the BD group. The lowest counts of ARGs were found in MBO group.

## Discussion

The results of this study demonstrates an increase biliary infection rate and antibody resistance of the pathogenic bacteria confirmed by culture-dependent method after PTBD; meanwhile, a stable microbiota in disease-free and malignant obstructive biliary tracts is detected, which used high-throughput sequencing approach, biliary microbiota dysbiosis present and antibiotic resistance genes rise after PTBD. This is the first study to attempt to describe the microbiota of the entire biliary system with no pathology aided by the support of the ischemia-free liver transplantation technique. While methods such as ANOVA, hierarchical clustering via heatmap, and PCoA analysis can identify differences of microbial components and diversity both between and within the disease-free, obstructive and drainage biliary tracts, culture-independent sequencing approach provides novel insight into drainage-associated biliary infection. Our study showed an association between biliary drainage and bacterial infection as well as antibiotic resistance of bacteria from the microbiome perspective.

The presence of microbiota in healthy bile ducts is not widely accepted. Under normal conditions, the continuous flushing action of bile and the bacteriostatic effects of bile salts were supposed to create a hostile environment for bacteria. In addition, the sphincter of Oddi acts as an anatomical barrier that protects the biliary tract from bacterial invasion (Sung et al., 1992). It has only recently become evident that the biliary system may harbor a complex microbiota that is most likely present in non-pathogenic situations. The composition of microbial profile in the gallbladder of healthy pig was presented, which showed that the gallbladder was mainly populated by members of the phyla Proteobacteria, Firmicutes, and Bacteroidetes (Jiménez et al., 2014). Molinero (Molinero et al., 2019) et al. discovered that bacterial communities were present in the bile of gallbladders with on pathology, and the microbiota composition differed significantly from the cholelithiasis patients. Under normal conditions, most of bile is synthesized in the liver, gathered through the bile ducts and eventually stored in the gallbladder (Strazzabosco and Fabris, 2008). Normal bile ducts contain only a small amount of bile, it is not feasible to collect bile from normal bile ducts by puncturing them. The technical and ethical challenges of bile sample collection in healthy individuals have limited study the normal biliary microbiome. Molinero (Molinero et al., 2019) et al. aimed to address the limitation by collecting 13 bile samples from the gallbladders of liver-transplant donors with no biliary pathology (control group), and comparing them with bile samples from the gallbladders of patients undergoing surgery for cholelithiasis. These pyrosequencing studies exclusively studied bile obtained from gallbladders. Our study collected not only four bile samples from the gallbladder aspiration directly during conventional liver transplantation, but also four bile samples collected from the common bile duct during ischemia-free liver transplantation while the liver was still continuously secreting bile. In our results, disease-free biliary tract is dominated by the bacterial sequences mainly assigned to the genus of *Acinetobacter*, *Burkholderia*, *Mycobacterium*, and *Nocardia*.

It is worth noting the characteristics of these four bacterial genera. The cell walls of *Mycobacterium* and *Nocardia* contain mycolic acids and wax-like lipids, forming a hydrophobic barrier that can resist bile acid toxicity and nutrient sequestration. This structural adaptation also could maintain cellular integrity in concentrated and acidic gallbladder bile. Metabolic flexibility of *Acinetobacter* allows utilization of limited carbon sources (e.g., acetate, lactate) under oligotrophic conditions, contributing to its ubiquity in biliary microbiota. *Burkholderia* genus upregulates nutrient transporters and stress-response pathways in low-nutrient niches, supporting persistence in bile with fluctuating nutrient availability. Collectively, these genera employ structural resilience (mycolic acids) and metabolic versatility (nutrient scavenging) to overcome biliary stressors, including nutrient limitation, bile acid exposure, thereby dominating the nutrient-deficient biliary microenvironment. Our results reveal that the entire biliary system harbors complex and stable microbiota, with the small intra-group variation in the control group. To the best of our knowledge, this is the first attempt to describe a complex microbiota in the whole biliary system without pathology. This result is critical for establishing healthy baselines from which to detect differences associated with diseases.

Patients with biliary tract malignancies are predisposed to develop biliary obstruction and obstructive jaundice that requires interventions including ERCP or PTBD (Sameer and Mishra, 2021). These interventions inadvertently introduce gut microbiota into the biliary tract. The overall infection rate related to these procedures exceeded 60%, with similar infection rates for the two procedures (Khardori et al., 1991; Turan et al., 2021). The most common manifestation was cholangitis, followed by bacteremia, with 8.2% of patients died after PTBD (Turan et al., 2021). Besides, biliary stenting has been shown to induce a significant alterations in bile microbiota, which is associated with a higher risk of postoperative surgical site infection (Scheufele et al., 2017).These findings support the notion that the introduction of bacteria into the biliary system during a biliary intervention may alter the bile microbial composition and led to infections. On the other hand, biliary tract microbial alterations can affect the tumor inhibitory effect of bile (Shrader et al., 2021). Although the increased incidence of infections following biliary drainage is a well-documented clinical phenomenon, there are limited studies investigating the relationship between biliary drainage and the overall biliary microbiota. In our study, the bile samples obtained from different categories of biliary tracts could be characterized by distinct compositions of metagenomic reads on high taxonomic (genus and species) levels. Our comparative analysis of deeply sequenced metagenomes revealed clear microbial patterns in biliary tracts with malignant obstruction, as well as those received drainage interventions. Although some rare species were defined as discriminatory features of the MBO group, it is challenging to establishing consistent relationships between specific taxa and biliary tract tumor or MBO. Our results demonstrated that the microbial composition of control subjects and those with MBO subjects were similar. The richness index indicated the population of bacteria in MBO group was less than the other two groups. These suggest that malignant obstruction of the biliary tract actually decreased the bacteria in the bile ducts. On the contrary, the BD operation resulted in a dramatic increase in bacterial population in the bile ducts. This provides an explanation for the clinical phenomenon that patients with malignant biliary obstruction after drainage are more susceptible to infection. Previous studies have shown similar results that no cases of cholangitis in malignant obstruction, except for those who have received prior instrumentation, and all patients with malignant obstruction who present with cholangitis have stents (Englesbe and Dawes, 2005).

Our results have shown that the composition of the biliary microbiota in BD group was quite different from that in the MBO group and control group. The predominant bacterial genera in the BD group were *Bacillus*, *Streptococcus*, *Staphylococcus*, and *Klebsiella*. This result is consistent with previous studies. In a retrospective study of 528 cases, the most common pathogens in bile cultures for postoperative biliary tract infections after PTBD were *Escherichia coli*, *Klebsiella pneumoniae*, *Enterococcus faecalis* and *Pseudomonas aeruginosa* (Xing et al., 2022). Biliary drainage leaded to the overgrowth of these bacteria at the expense of a large number of otherwise benign bacteria. Notably, these bacteria could represent an important reservoir for new infections in patients who were chronic carriers of them. It has been reported that bile microbiota is fundamentally different in patients with periampullary carcinoma treated with preoperative BD than those who are not treated with biliary drainage, which contributed a shift of the biliary microbiome from *Escherichia coli* in non-stented patients towards increased contamination with *Enterococcus faecalis* and *Enterobacter cloacae* (Scheufele et al., 2017). In our study, bacterial infections were detected in 70.4% of the BD group samples and 13.8% of the MBO group samples by culture-dependent method. It has been a clinically accepted concept that biliary drainage induces a shift in the bile microbiota towards a more aggressive behavior. In clinical practice, 76% of patients still develop biliary-drainage-related infections after prophylaxis with antibiotics (Khardori et al., 1991). Notably, BD group exhibited significantly higher relative abundances of *Mycobacteria* and *Nocardia* compared to the MBO group. While these genera are uncommon in typical biliary tract infections, atypical *Mycobacterial* infections frequently occur in immunocompromised individuals or those with chronic respiratory disease, and approximately 60% of *Nocardiosis* cases are linked to preexisting immune compromise (Sharma and Upadhyay, 2020; De Benedetto et al., 2022). The potential occurrence of these genera raises questions about whether they can serve as evidence of underlying immune dysfunction, though this remains unknown. Furthermore, the *Mycobacterium* genus primarily includes *Mycobacterium tuberculosis* and *non-tuberculous Mycobacteria* (NTM) species. The predictive value of this culture-independent method for tuberculosis in patients who have undergone biliary drainage for malignancy remains unclear. Immunocompromised individuals are generally considered at increased risk for developing active tuberculosis. However, in low-tuberculosis-incidence European countries, immunocompromised patients have a relatively low risk of active tuberculosis unless there is a clear history of exposure to *M. tuberculosis* and ongoing HIV replication with low CD4 counts (Sester et al., 2025). To date, tuberculosis detection tests, including widely used immunological assays such as the tuberculin skin test (TST) and interferon-γ release assays (IGRA), have demonstrated poor predictive value for incident tuberculosis in immunocompromised individuals (Sester et al., 2025). Similarly, this culture-independent method cannot distinguish between latent and active tuberculosis, nor can it differentiate between long-term and recent infections. In contrast, China is a region with high tuberculosis incidence, accounting for the third largest share of global tuberculosis cases (Cui et al., 2020). Reports estimate that there were 780,000 new cases in 2021. The latent tuberculosis infection rate among individuals aged 15 and above is 20.3%, and it shows an increasing trend with age (Cui et al., 2020). Ongoing transmission and new exposure to *M. tuberculosis* are common in China, and the risk factors for tuberculosis differ from those in Europe. In China, younger age, larger family size, and presence of new cases are associated with higher odds of recent transmission, while higher education (university and above) and occupation as non-physical workers are identified as protective factors (Liu et al., 2024). Therefore, the increased abundance of *Mycobacterium* in the BD group cannot be solely attributed to a decline in immune function; it may also be associated with additional factors related to tuberculosis exposure following biliary drainage. Further research is needed to comprehensively and objectively interpret this finding.

To gain further insight into microbiological features of abnormal biliary tracts, we subjected each bile sample to culture and resistance study. Although not all pathogens can be cultured, the antibiotic resistance results allow us to infer genotypes of clinical importance. Genes associated with antibiotic resistance, including those identified through sequence homology (Zhang et al., 2021), are widespread in bacteria and play roles in various biological processes, such as efflux systems and cell-to-cell communication (Blair et al., 2015). Resistance mechanisms of biliary tract bacterial community are similar to the conventional ones known clinically, such as antibiotic efflux, antibiotic inactivation and alteration of antibiotic target (Blair et al., 2015). The presence of the ARGs underlined the strong impact of BD procedures on bile microbiota. The highest portion of ARGs was present in the BD group, especially in patients who received stent intervention. Bacteria gene in the BD group encode mostly efflux-pump proteins. The most frequent ARGs in the biliary tract encode one of the families of efflux pump proteins associated with multidrug resistance: the resistant nodule division (RND) family. The RND-family efflux pumps are associated with clinically significant drug resistance in gram-negative bacteria (Laura and Piddock, 2006). Several studies have also shown a role of the RND family of efflux pumps in the pathogenicity of these bacterial species. For example, *Pseudomonas aeruginosa* lacking the RND-family efflux pump failed to kill leukocyte-deficient mice, whereas the parent strain of this mutant *P. aeruginosa* caused a fatal infection (Hirakata et al., 2002). Besides, bacteria expressing multidrug-resistance (MDR) efflux pump proteins can produce bile resistance that allows bacteria to survive in certain ecological niches alongside bile salts (Laura and Piddock, 2006). Our results suggested that biliary drainage appears to induce a shift in bile microbiota, making it more aggressive and resistant to antibiotics and bile. Clinically, the majority of human-associated and mobile ARGs were already present in ESKAPE pathogens (i.e., *Enterococcus faecium*, *Staphylococcus aureus*, *Klebsiella pneumonia*, *Acinetobacter Baumannii*, *Pseudomonas aeruginosa*, and *Enterobacter* sp*ecies*) (Zhang et al., 2021). Local epidemiology, including geographic and hospital-specific factors, likely influences the composition of the biliary microbiota. According to the 2014–2019 surveillance data on bile bacterial resistance from the National Bacterial Resistance Surveillance Network of China and related literature reports, recent epidemiological characteristics of bacterial infections in biliary surgery are as follows: The bacterial flora distribution in biliary tract infections is predominantly Gram-negative, accounting for approximately 70%. The top five pathogens are *Escherichia coli* (30.90%), *Klebsiella pneumoniae* (12.70%), *Pseudomonas aeruginosa* (4.90%), *Enterobacter cloacae* (4.50%), and *Acinetobacter baumannii* (2.20%). Gram-positive bacteria account for about 30%, with Enterococcus species being the predominant pathogen (China, 2021). These bacteria are considered to originate from the intestine tract and may exert a pathogenic effect in the presence of bile duct compression. In our study, extrapolation to antibiotic-resistance phenotypes inferred from biomarker sequences were validated by traditional microbiology approaches, suggesting that bacterial drug resistance encoded after BD procedures was likely associated with the unique microbial profile. Our results also suggested that biliary drainage contributes to the development of multidrug-resistant (MDR) bacteria. Due to the relief of biliary obstruction, some bile can re-enter the intestines, the intestinal bacteria may develop both bile resistance and antibiotic resistance after bile re-exposure. Finally, these bacteria could easily colony in the biliary tract through bacterial translocation.

There are several limitations in our study. First, the sample size was relatively small, which may limit the generalizability of the findings. A larger cohort would provide more robust data and allow for more precise conclusions. Second, the duration of post-drainage observation varied among patients, introducing potential variability in the outcomes, such as environmental contaminant. Additionally, while metagenomic analysis provided valuable insights, it might not fully capture the functional activities of the microbial communities, necessitating complementary approaches such as transcriptomics or metabolomics for a more comprehensive understanding. Despite these limitations, this study lays the groundwork for future investigations into the complex interactions between biliary drainage, microbial shifts, and antibiotic resistance development. Further research is needed to explore the long-term implications of these findings and their clinical relevance in managing biliary tract infections.

In conclusion, our study demonstrated that a distinct taxonomic composition were found in the bacterial communities of the disease-free, malignant obstructive and post-drainage biliary tracts. To the best of our knowledge, this study is the first to use metagenomics to characterize bile microbial communities of the who bile ducts with no pathology and bile ducts that have received biliary drainage procedure. An abundance bacteria were found in the biliary duct with no pathology, and the composition of the microbiota was similar to that of the biliary microbiota of patients with MBO who did not received biliary drainage. BD procedures might alter the composition of bile microbiota, leading to a reduction in biliary microbial diversity. Our results also showed that biliary drainage was associated with increased diversity of antibiotics drug resistance genes. The data obtained in this initial study represent a baseline metagenomic characterization of normal biliary microbiota. The results could provide new information about biliary tract infections associated with BD and new evidence to support that BD induces a shift in bile microbiota to be more aggressive and resistant to antibiotics.

## Data Availability

The datasets generated for this study can be found in the SRA database under the accession number: PRJNA1000238 [https://www.ncbi.nlm.nih.gov/sra/PRJNA1000238].
