# Supplementary material for: Biliary microbiota in disease-free, obstructive and post-drainage biliary tracts
Source: Front Cell Infect Microbiol. 2025 Dec 10;15:1674341. doi: 10.3389/fcimb.2025.1674341 (PMC12727556; doi:10.3389/fcimb.2025.1674341)
Supplement: Supplementary file 1 [file Table1.docx]

**Supplemental information**

**Table S1. Clinical context of MBO and BD group.**

|  |  | MBO | BD | P value |
| --- | --- | --- | --- | --- |
| Age(year) |  | 58.4 | 49.9 | **0.039** |
| Gender | Male | 19 | 19 |  |
|  | Female | 10 | 7 | 0.698 |
| Underlying malignancies | ICC | 17 | 14 |  |
|  | HCC | 6 | 4 |  |
|  | Pancreatic Cancer | 4 | 6 |  |
|  | Liver Metastases | 2 | 3 | 0.773 |
| Bile culture | Positive | 4 | 19 |  |
|  | Negative | 25 | 8 | **0.000** |
| Blood culture | Positive | 0 | 6 |  |
|  | Negative | 7 | 10 |  |
|  | None | 22 | 11 | 0.074 |
| Antibiotic therapy | Yes | 9 | 16 |  |
|  | No | 20 | 11 | **0.031** |
| Infection ^a^ | Yes | 9 | 21 |  |
|  | No | 20 | 6 | **0.000** |
| Fever | Yes | 4 | 14 |  |
|  | No | 25 | 13 | **0.004** |
| Inflammatory | Yes | 20 | 21 |  |
|  | No | 9 | 6 | 0.330 |
| Jaundice ^b^ | Yes | 28 | 21 |  |
|  | No | 1 | 6 | **0.041** |
| Liver dysfunction ^c^ | yes | 29 | 23 |  |
|  | no | 0 | 4 | **0.048** |
| Cholangiectasis | Yes | 22 | 14 |  |
|  | No | 0 | 4 | **0.009** |
| Obstructive sited | Distal | 7 | 7 |  |
|  | Proximal | 22 | 20 | 0.560 |

1. positive for bile or blood culture ; or need antibiotics therapy.

b.T-Bil ≥2 (mg/dl).

c. ALP (IU) >1.5 STD;cGTP (IU) >1.5 STD;AST (IU) >1.5 STD;ALT (IU) >1.5 STD.

**Supplementary figures**


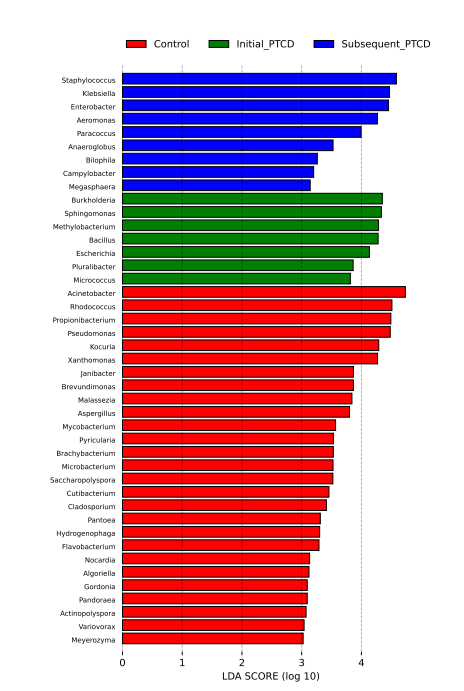


### **Fig. S1** Distinctive taxa of bile sample groups LEfSe analysis at the genus level among control (red) group, MBO (green) group and BD (blue) group.

**Analysis of microbial communities in species level**

**
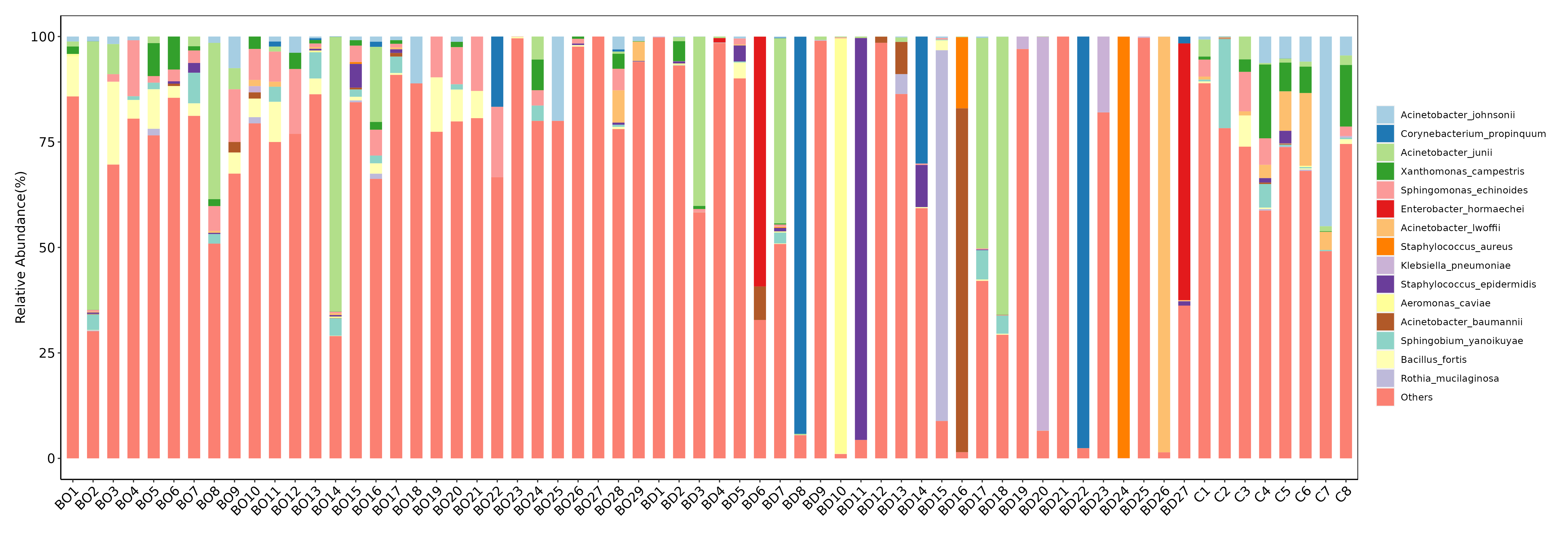
**

**Fig. S2** The top 15 species and composition of each samples

**Fig. S3 the metabolic pathway predictions of genome levels**


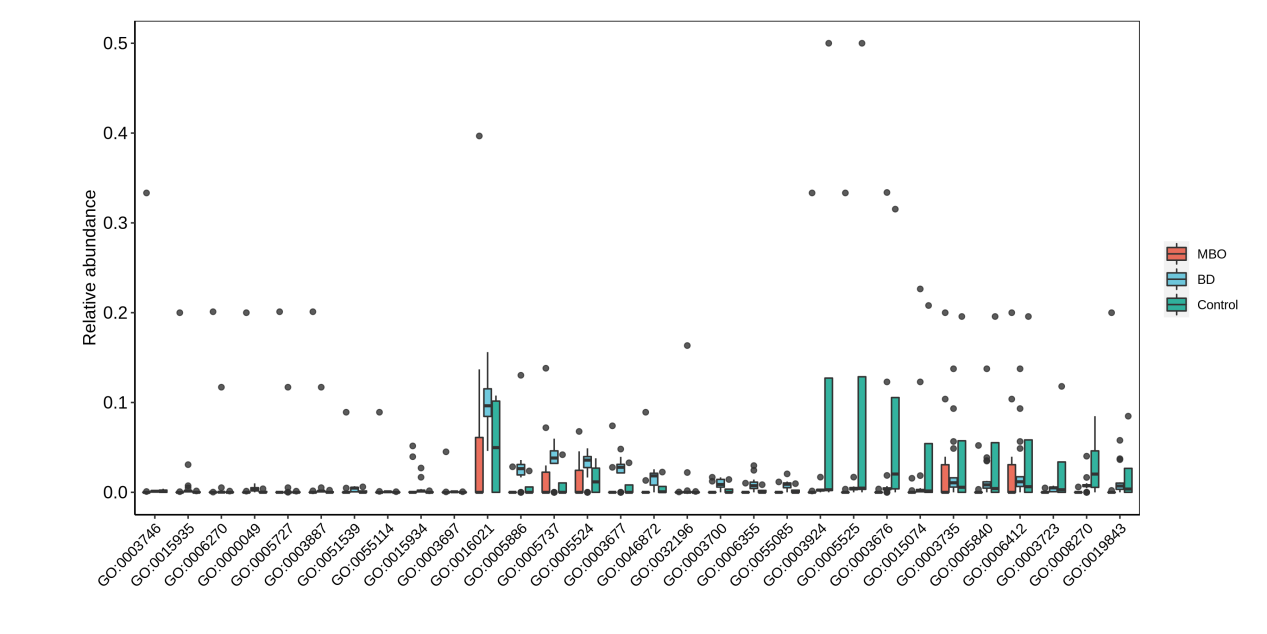
**A**

1. The relative abundance of GO pathway related to cellular componet (including integral compent of menbrane, cytoplasm and plasma menbrane) and molecular function (including ATP binding, DNA binding and metal ion binding) in BD group were higher than that in MBO and control group.The relative abundance of reads annotated about molecular functions of GTP, nucleic acid, RNA and zinc ion binding, biological process of DNA integration and translation were higher in control group.

**B**


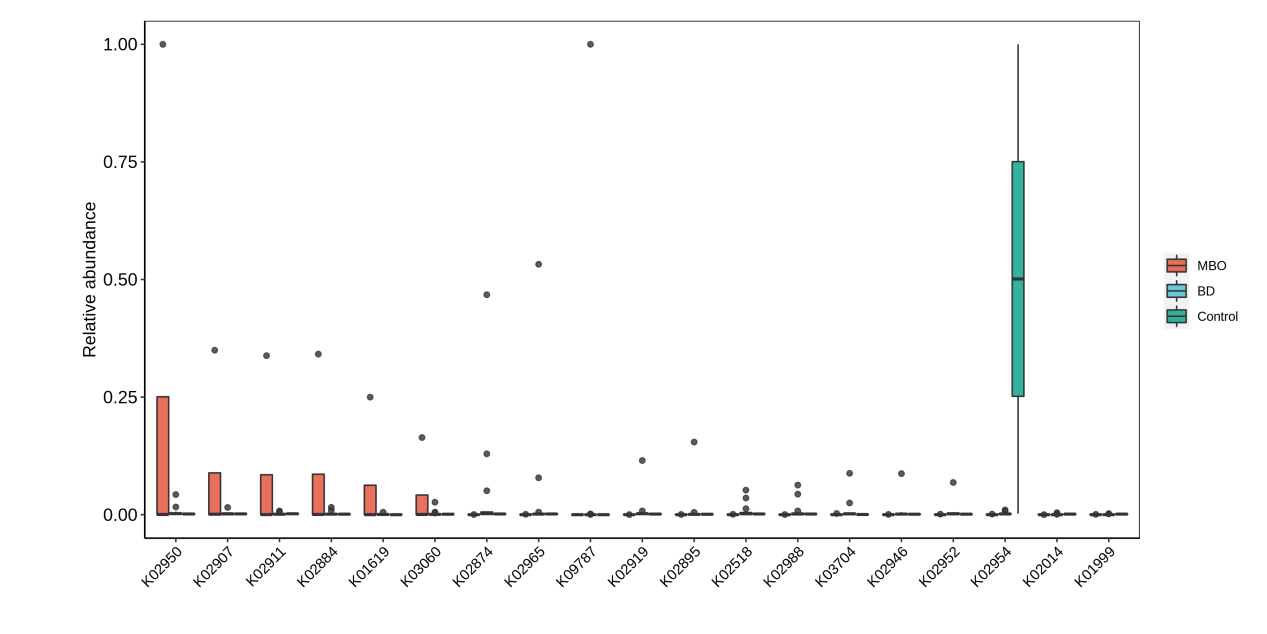


1. Different distribution of KEGG pathway among three groups; K02954 and K02950: small subunit ribosomal protein; K02907, K02911 and K02884: large subunit ribosomal protein; K03060: DNA-directed RNA polymerase subunit omega.

**C**


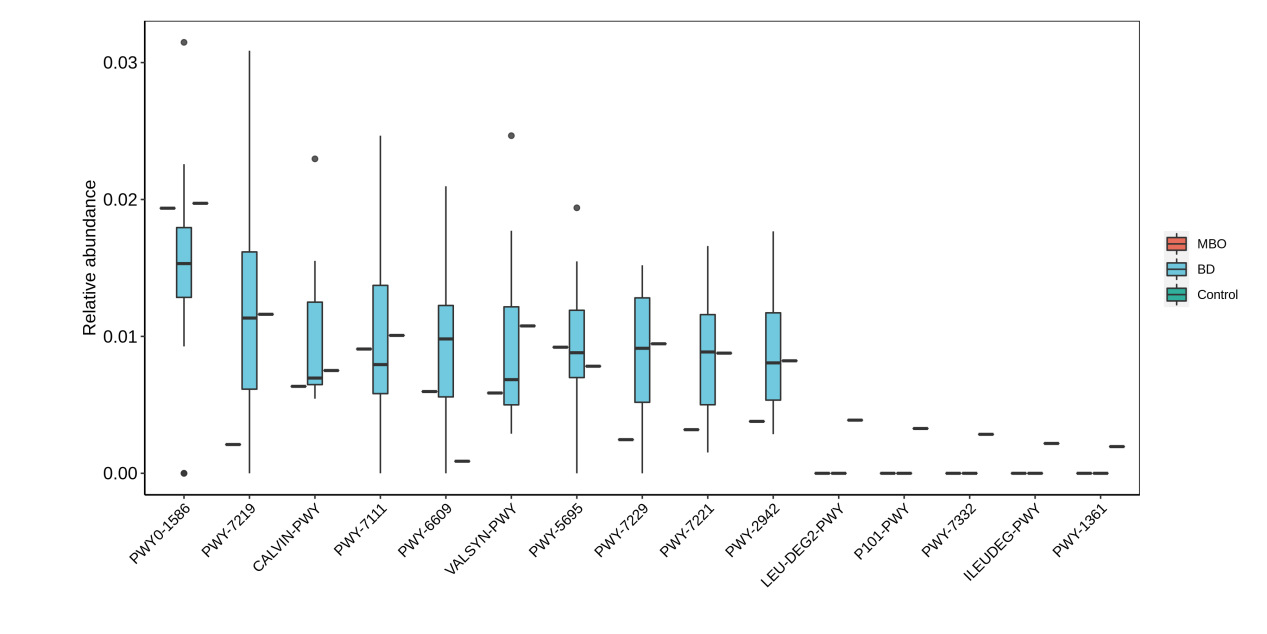


1. MetaCyc pathway predictions among three group. Higher relative abundances for peptidoglycan maturation, [nucleoside and nucleotide biosynthesis](https://biocyc.org/META/NEW-IMAGE?type=ECOCYC-CLASS&object=Nucleotide-Biosynthesis), [amino acid biosynthesis](https://biocyc.org/META/NEW-IMAGE?type=ECOCYC-CLASS&object=Amino-Acid-Biosynthesis), [carbohydrate biosynthesis](https://biocyc.org/META/NEW-IMAGE?type=ECOCYC-CLASS&object=Carbohydrates-Biosynthesis) were found in BD group. PWY0-1586:peptidoglycan maturation (meso-diaminopimelate containing). PWY-7219; adenosine ribonucleotides de novo biosynthesis; PWY-7111: pyruvate fermentation to isobutanol (engineered); CALVIN-PWY: Calvin-Benson-Bassham cycle; PWY-5695: inosine 5'-phosphate degradation; PWY-6609: adenine and adenosine salvage III. VALSYN-PWY:L-valine biosynthesis; PWY-7221:guanosine ribonucleotides de novo biosynthesis; PWY-2942: L-lysine biosynthesis III; LEU-DEG2-PWY: L-leucine degradation I; P101-PWY:ectoine biosynthesis; PWY-7332:superpathway of UDP-N-acetylglucosamine-derived O-antigen building blocks biosynthesis; ILEUDEG-PWY:L-isoleucine degradation I; PWY-1361:benzoyl-CoA degradation I (aerobic).

**Procedure for Bacterial Culture and Antimicrobial Susceptibility Testing of Bile Samples Using VITEK®2 System**

1. Bacterial Culture

1.1 Sample Processing: Bile samples (1-2 mL) were collected under sterile conditions and immediately inoculated onto blood agar, MacConkey agar, and chocolate agar plates. Plates were incubated aerobically at 37°C for 18-24 hours, followed by anaerobic incubation for 48 hours to isolate facultative and anaerobic bacteria.

1.2 Pure Culture Isolation: Distinct colonies were subcultured to obtain pure isolates, which were identified by Gram staining and preliminary biochemical tests before VITEK®2 analysis.

2. VITEK®2 System Operation

2.1 Instrument Initialization: The VITEK®2 Compact system was powered on sequentially (stabilized power supply → UPS → printer → terminal → reader → computer). After system startup, the incubator temperature was verified to reach 35±1°C, and the status indicator showed "OK".

2.2 Bacterial Suspension Preparation: Pure colonies (18-24 hours old) were suspended in 3 mL of 0.45% sterile saline to achieve a turbidity of 0.5-0.63 McFarland standard using a nephelometer.

2.3 Test Card Loading: Identification (ID) and antimicrobial susceptibility testing (AST) cards were loaded onto the card carrier, with AST cards paired with corresponding ID cards. The sample tube was placed in the suspension port, and barcodes of the carrier and cards were scanned into the VITEK®2 software. Patient information and sample details were recorded in the system.

2.4 Card Incubation and Reading: The card carrier was inserted into the filling station for automated inoculation (≈70 seconds). After filling, carriers were transferred to the incubation chamber, where the system continuously monitored and analyzed growth patterns every 15 minutes. Results were automatically generated when sufficient growth was detected (5-18 hours).

3. Antimicrobial Susceptibility Testing

3.1 AST Card Selection: AST-N248 or AST-GN16 cards were used for Gram-negative bacteria, while AST-P609 cards were used for Gram-positive isolates, containing antibiotics relevant to biliary tract infections (e.g., ceftriaxone, ciprofloxacin, meropenem).

3.2 Interpretation Standards: Minimum inhibitory concentrations (MICs) were interpreted according to Clinical and Laboratory Standards Institute (CLSI) M100-Ed33 and European Committee on Antimicrobial Susceptibility Testing (EUCAST) v13.0 breakpoints. Results were categorized as susceptible (S), intermediate (I), or resistant (R).

4. Quality Control

Reference strains (Escherichia coli ATCC 25922, Pseudomonas aeruginosa ATCC 27853, Staphylococcus aureus ATCC 29213) were tested weekly to validate system performance. Internal controls within each AST card were monitored to ensure proper incubation and reagent reactivity.

5. Result Reporting

Final reports included bacterial identification (genus and species), MIC values, and interpretive categories. Resistance phenotypes (e.g., extended-spectrum β-lactamase producers) were flagged based on CLSI/EUCAST criteria for clinical decision-making.
